# Characterization and Long-Term Prognosis of Patients with Different Phenotypes of Dilated Cardiomyopathy

**DOI:** 10.3390/jcdd11070220

**Published:** 2024-07-12

**Authors:** Shuyuan Zhang, Shiqi Gao, Zhuang Tian, Shuyang Zhang

**Affiliations:** State Key Laboratory of Complex Severe and Rare Diseases, Department of Cardiology, Peking Union Medical College Hospital, Chinese Academy of Medical Sciences & Peking Union Medical College, Beijing 100730, China; 18629952005@163.com (S.Z.); gsq_pumch@163.com (S.G.)

**Keywords:** dilated cardiomyopathy, left ventricular hypertrabeculation, prognosis, heart failure

## Abstract

Background: Long-term prognosis of dilated cardiomyopathy (DCM) in the Chinese population is lacking, and the left ventricular (LV) hypertrabeculation phenotype usually overlaps with DCM. Objectives: The study aims to investigate whether the presence of the LV hypertrabeculation phenotype confers additional adverse prognostic information for DCM patients. Methods: We retrospectively reviewed all DCM patients (≥18 years of age at diagnosis) hospitalized in the Peking Union Medical College Hospital between September 2002 and September 2022. The eligible patients were divided into two groups based on echocardiography at diagnosis: the isolated DCM (*n* = 353), and DCM with the LV hypertrabeculation phenotype (*n* = 97). The primary endpoint was major adverse cardiac events (MACEs), and multivariate Cox hazards regression models were used to compare the endpoints between the two groups. Results: During a mean follow-up time of 4.6 years, there was no significant difference in the primary endpoint between the isolated DCM and DCM with the LV hypertrabeculation phenotype (*p* = 0.19). The risk of MACEs in the first 5 years was significantly higher in DCM with the LV hypertrabeculation phenotype than isolated DCM (adjusted HR [95%CI]: 1.83 [1.21–2.77]) and after 5 years the effect of the LV hypertrabeculation phenotype as a prognostic attenuated. Subgroup analysis found a significant interaction for the incidence of MACEs between sex and DCM subtypes (*p* for interaction = 0.01). Conclusions: DCM with LV hypertrabeculation phenotypes had a higher early (first 5 years) risk of MACEs. For males, the presence of LV hypertrabeculation phenotypes might be an important clue for identifying high-risk DCM patients.

## 1. Introduction

Dilated cardiomyopathy (DCM) is a kind of heterogeneous disease characterized by left ventricular (LV) enlargement and systolic dysfunction, caused by a combination of environmental modifiers and underlying genetic susceptibility [1]. Approximately 1/250 people will develop DCM, placing a growing medical and economic burden on healthcare systems [1]. Despite advances in primary prevention and treatment for DCM, the prognosis of DCM is poor, with a 10-year survival rate of approximately 63% [2]. Even with the use of guideline-directed medical therapy for heart failure (HF), more than 77% of DCM patients who experience HF die within 2 years of diagnosis, mostly owing to sudden cardiac death and systemic embolism [3].

According to the 2023 European Society of Cardiology (ESC) guidelines, LV hypertrabeculation is not a distinct type of cardiomyopathy; instead, it should be considered a morphologic trait shared by many phenotypically different cardiomyopathies [4]. A report on genetic variants identified in left ventricular noncompaction (LVNC) patients supported this point because most variants were in sarcomeric genes associated with hypertrophic cardiomyopathy and DCM [5]. In a retrospective analysis of Chinese adults with LVNC, our team found that LVNC patients with dilated phenotype had a significantly higher risk of major adverse cardiovascular events (MACEs) than those with isolated LVNC [6]. For patients with LVNC < 18 years of age, baseline LV dilation and systolic dysfunction are associated with progression to death or heart transplantation [7]. Sedaghat-Hamedani et al. [8] found that the risk of MACEs was significantly higher in LVNC cases compared with a cohort of age-matched DCM patients. It is unclear whether the presence of the LVNC phenotype confers additional adverse prognostic information for DCM patients. Evidence regarding the characteristics, evolution, and outcomes of DCM patients in the Chinese population is lacking. Therefore, we aimed to characterize DCM patients at baseline, describe their natural history, explore risk factors, and compare the prognosis of isolated DCM with those of the LV hypertrabeculation phenotype.

## 2. Methods

### 2.1. Study Design and Patients

This was a single-center retrospective cohort study, and all DCM patients eligible for inclusion were analyzed. Enrolled DCM patients (≥18 years of age at diagnosis) were all hospitalized in the Department of Cardiology, Peking Union Medical College Hospital (PUMCH) between September 2002 and September 2022. Data were collected from the electronic medical record system of PUMCH. The diagnosis of DCM was defined as the presence of LV dilatation and systolic dysfunction with left ventricular ejection fraction (LVEF) < 50% [4,9]. We excluded individuals with significant coronary artery disease (>50% stenosis of a major coronary artery), a history of uncontrolled systemic hypertension (>160/100 mmHg), alcoholic cardiomyopathy (>80 g/d for more than 5 years), valvular heart disease, congenital heart disease, pericardial diseases, or active myocarditis. A total of 34 DCM patients who had missing clinical data were excluded, and 450 eligible patients were included in this analysis. We divided DCM patients into two groups based on baseline echocardiography at diagnosis: (1) isolated DCM, and (2) DCM with the LV hypertrabeculation phenotype, which was diagnosed as a two-layered structure of the myocardium with a ratio of non-compaction to compaction of ≥2:1 at end-systole according to the Jenni criteria for echocardiographic diagnosis [10].

The study protocol was approved by the ethical review board of PUMCH, Chinese Academy of Medical Sciences, Beijing, China (protocol code I-23PJ040). All patients in PUMCH gave their written informed consent.

### 2.2. Data Collection

Baseline characteristics were collected for all patients, including clinical assessment, laboratory examinations (lipid profile, creatinine, and N-terminal pro-b-type natriuretic peptide [NT-proBNP]), electrocardiography, echocardiography, and medications (angiotensin-converting enzyme inhibitor/angiotensin receptor blocker/angiotensin receptor/neprilysin inhibitor, β-blockers, diuretics, statins, antiplatelet agents, and anticoagulant agents). Echocardiography measurements were performed according to current international guidelines [11]. In particular, LVEF was calculated using the Simpson biplane method. Mitral regurgitation (MR) severity was assessed using a multiparametric approach by calculating the effective regurgitant orifice area as determined by the proximal iso-velocity surface area method [12].

### 2.3. Definitions of Clinical Endpoints

All patients were followed up via telephone interviews or clinic visits. Investigators underwent training for standard follow-up information collection to obtain high-quality data. Follow-up ended on 31 December 2023, or at the time of death or the first occurrence of primary endpoints. The primary endpoints in this study were MACEs, which is a composite of HF events, severe ventricular arrhythmias, left ventricular assist device implantation, and any heart transplantation. Severe ventricular arrhythmias were defined as sustained ventricular tachycardia (≥30 s), ventricular fibrillation, appropriate implantable cardiac-defibrillator therapy, or sudden cardiac death [13]. The secondary endpoint included the first occurrence of HF events, severe ventricular arrhythmias, left ventricular assist device implantation, heart transplantation, CV death, and all-cause death. 

### 2.4. Statistical Analysis

The clinical characteristics were compared for categorical variables using chi-square tests or the Fisher exact test (expressed as numbers [percentages]), and for quantitative variables using the Student *t*-test (expressed as mean ± standard deviation [SD]) or the Mann–Whitney *U* test (expressed as median [interquartile range]) as appropriate. Cause-specific univariate and multivariate Cox hazards regression models were used to analyze the risk factors associated with MACEs, and the hazard ratios (HRs) and 95% confidence intervals (CIs) were calculated. Multivariate Cox hazards regression models were used to compare the endpoints between two groups using a backward procedure, which included two models to control for confounding factors. Model 1 was adjusted for age and sex; model 2 was further adjusted for body mass index (BMI), New York Heart Association (NYHA) functional class, medical history of hypertension and diabetes, ST-T changes, moderate to severe MR, and Ln(NT-proBNP). Kaplan–Meier (KM) curves were generated for MACEs and HF to compare groups using a log-rank test during the first 5 years of follow-up and using Cox regression models, including an interaction term for time to estimate separate HRs to compare isolated DCM and DCM with LV hypertrabeculation phenotype groups during the first 5 years and over 5 years of follow-up. Subgroup analyses were conducted based on sex, age (<60 or ≥60 years old), NYHA functional class (I/II or III/IV), and LVEF (<40% or 40–50%), and tests for interaction were also performed. The statistical analysis used R Version 4.0.2 (R Core Team, Vienna, Austria). A two-sided *p* value of <0.05 was considered statistically significant.

## 3. Results

### 3.1. Baseline Characteristics of DCM Patients

A total of 450 DCM patients were included in this study, with an average age of 48.1 ± 16.0 years, and 305 (67.8%) were male. Significant differences were found for BMI (*p* = 0.001), LVEF (*p* = 0.002), interventricular septum (*p* = 0.03), and Ln(NT-proBNP) (*p* = 0.02) between included and excluded patients (Table S1). Among the included patients, 353 (78.4%) patients were in the isolated DCM group and 97 (21.6%) were in the DCM with LV hypertrabeculation group. Isolated DCM patients had a significantly higher BMI as well as the proportion of ST-T changes and a history of hypertension or diabetes, compared with patients who had DCM with LV hypertrabeculation (Table 1). There were no significant differences in echocardiography features, laboratory examinations, and medications between the isolated DCM and DCM with the LV hypertrabeculation phenotype.

### 3.2. Association of Cardiovascular Risk Factors with MACEs among DCM Patients

During a mean follow-up time of 4.6 years, 244 (54.2%) DCM patients underwent MACEs, including 204 with heart failure, 91 with cardiovascular death, 27 with major ventricular arrhythmias, 11 with cardiac resynchronization therapy, and nine with heart transplantation (some participants had more than one component event). We conducted a multivariable model for MACEs, based on the univariable analysis reported in Table 2, which revealed the presence of moderate or severe MR (HR [95%CI], 1.53 [1.12–2.08]; *p* = 0.01) and increasing Ln(NT-proBNP) (1.28 [1.193–1.44]; *p* < 0.001) as independent predictors of MACEs for DCM patients at baseline. 

### 3.3. Comparison in the Incidence of Clinical Outcomes between Different DCM Subtypes

**Figure 1:**
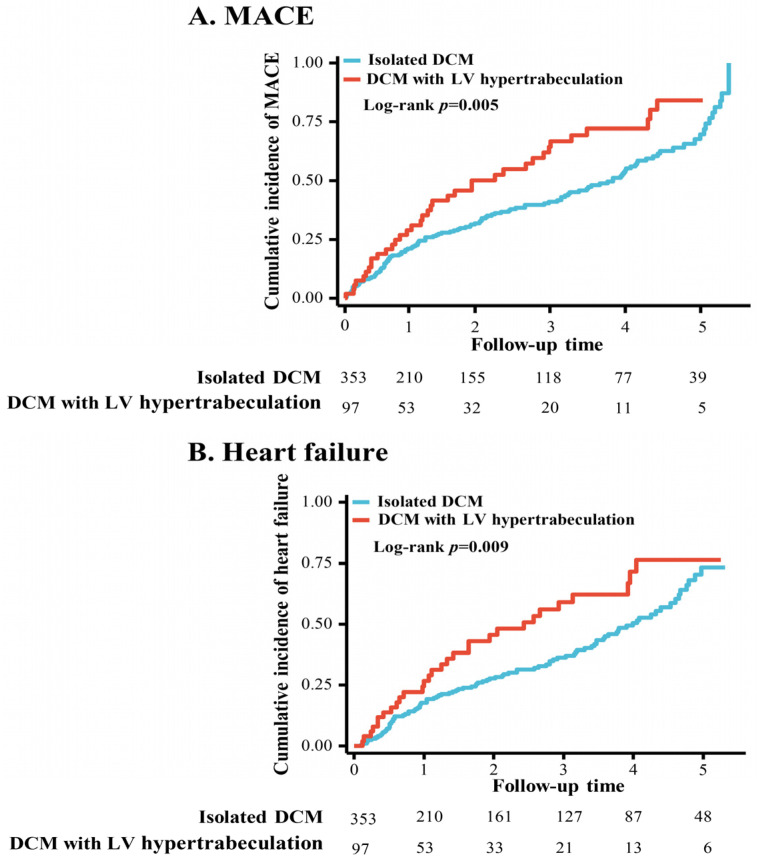
Cumulative incidence of MACEs by different subtypes of dilated cardiomyopathy over 5 years of follow-up. Abbreviations: MACEs, major adverse cardiovascular events; DCM, dilated cardiomyopathy; LV, left ventricular.

As shown in Table 3, multivariate Cox hazards regression analysis showed that there were no significant differences in MACEs, HF, major ventricular arrhythmias, cardiac resynchronization therapy, heart transplant events, all-cause death, and cardiovascular death between the isolated DCM and DCM with LV hypertrabeculation groups (all *p* > 0.05). The KM-estimated 5-year MACE proportions were 24.2%/100 person-year in the isolated DCM group vs. 40.7%/100 person-year in the DCM with LV hypertrabeculation group (*p* = 0.005) (Figure 1A). After adjustment for covariates, including age, sex, BMI, NYHA functional class, medical history of hypertension and diabetes, ST-T changes, moderate to severe MR, and Ln(NT-proBNP), the increased risk of MACEs (first 5 years) in DCM with the LV hypertrabeculation phenotype was even more pronounced (adjusted HR [95%CI]: 1.83 [1.21–2.77]) (Table S2). These differences were driven mainly by the rate of HF events (Figure 1B); however, after adjustment for related covariates, there was no significant difference in the rate of HF events between the two groups during the first 5 years (Table S2). After 5 years, the effect on the prognosis of DCM patients with the LV hypertrabeculation phenotype was attenuated (for MACEs, adjusted HR [95%CI]: 1.51 [0.80–2.84]) (Table S2).

### 3.4. Subgroup Analysis

**Figure 2:**
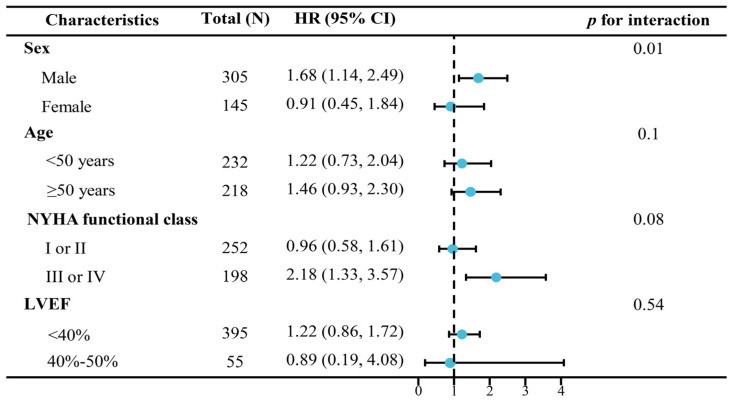
Subgroup and interaction analysis between the different subtypes of dilated cardiomyopathy and long-term prognosis of MACEs. Abbreviations: MACEs, major adverse cardiovascular events; HR, hazard ratio; CI, confidence interval; NYHA, New York Heart Association; LVEF, left ventricular ejection fraction. The dots in the figure represent the values of HRs.

The association between different DCM subtypes and MACEs was examined in subgroup analysis. A significant interaction was found between sex and DCM subtypes for the incidence of MACEs (*p* for interaction = 0.01). We also found that DCM with the LV hypertrabeculation phenotype showed a significantly higher risk of MACEs than isolated DCM among males (adjusted HR [95%CI]: 1.68 [1.14–2.49]). Although no interaction was found between NYHA functional class and different DCM subtypes for the incidence of MACEs in Cox hazards regression analysis (*p* = 0.08), statistical significance was observed among DCM patients with NYHA functional class III or IV (Figure 2).

## 4. Discussion

The LV hypertrabeculation phenotype can overlap with other types of cardiomyopathy, and DCM patients might present with morphological findings of LV hypertrabeculation. In our cohort, approximately 22% of all DCM patients were diagnosed with LV hypertrabeculation, and it is unclear whether the presence of LV hypertrabeculation is associated with poor prognosis for DCM patients. We analyzed a cohort of adult DCM patients with isolated DCM or DCM with the LV hypertrabeculation phenotype to identify risk factors for MACEs. Our main finding was that the presence of the LVNC phenotype for DCM patients was associated with a higher risk of MACEs and HF events in the early natural history (first 5 years); however, there was no significant difference in the risk of MACEs between the two groups during long-term follow-up. 

The classification and phenotypic definition of LVNC remains controversial. The American College of Cardiology guidelines classify LVNC as a hereditary cardiomyopathy [14], whereas the ESC guidelines suggest that LVNC should be categorized as a morphologic trait [4]. We observed significant differences regarding the primary endpoint of MACEs or HF events in the first 5 years, with most events occurring among adult patients with DCM, which is in agreement with previous studies. Sedaghat-Hamedani et al. [8] reported that a comparison between the LVNC cohort and an age-matched DCM cohort showed significantly higher rates of HF-associated events in LVNC. Meanwhile, several studies also found that LVNC with a dilated phenotype is associated with worse short-term outcomes for children [15,16]. Cardoso et al. [7] recently compared the outcomes of pediatric (<18 years old) DCM and DCM with LVNC phenotype patients. Their findings showed little consistency in that the 5-year composite event rates of death or heart transplant were, respectively, 57.6% and 57%, with most events occurring within 2.5 years after diagnosis. In our study, the 5-year composite event rate observed in the isolated DCM and DCM with LV hypertrabeculation phenotype groups was 34.2% and 39.2%, respectively. These findings reveal differences in the natural history of DCM between children and adults, and the prognosis of pediatric DCM with LV hypertrabeculation patients are worse than adults.

In our cohort, we compared the long-term prognosis of isolated DCM and DCM with LV hypertrabeculation phenotype patients and found no significant differences between the two groups in the composite of the primary endpoint or each component. These findings are in line with those reported by Towbin et al. [17] in a recent study that summarized the clinical features of different LVNC subtypes and found no significant difference in cardiovascular (CVD) events between the isolated LVNC and LVNC with dilated phenotype group. In our study, it may be explained by cardiac function in that no significant differences in NYHA functional class, LVEF, and NT-proBNP obtained between the two groups. However, we cannot ignore the differences in genetic background or underlying pathogenic variants in the two groups. Future studies should focus on the characterization of genetic findings and validating these in larger studies.

Our study suggested that compared with isolated DCM patients, the presence of LV hypertrabeculation is associated with a worse prognosis amongst males. Our results are also consistent with Cannata et al. that among DCM patients, male patients have been confirmed as independently associated with adverse prognosis even after the left ventricular reverse remodeling is achieved [18]. Several possible explanations have been offered as follows: firstly, sex hormones, such as estradiol, could protect against CVD in pre-menopausal women [19]. Increased levels of estradiol reduced myocyte apoptosis in an in vitro model of arrhythmogenic cardiomyopathy [20]. Then, a sex disparity in the prevalence of replacement fibrosis in DCM shows a higher prevalence in males than in females [21]. Finally, sex differences in gene expression in DCM patients may be responsible for differences in outcomes [22]. Truncating mutations in titin are thought to make individuals susceptible to developing contractile impairment, and men with such variants have been shown to have worse outcomes than women [23].

Our findings show that the presence of moderate to severe MR and elevated NT-proBNP emerged as the strongest predictors of MACEs among DCM patients. In our cohort, nearly 33.3% and 90% of DCM patients had moderate to severe MR and an elevated level of NT-proBNP, respectively. Plasma BNP levels have been widely shown to reflect disease severity by providing hemodynamic and prognostic information in HF patients of all etiologies [24]. As previously reported in patients with non-ischemic dilated cardiomyopathy, the severity of regurgitation is also an independent predictor of mortality and hospitalization [25].

This study has several limitations. First, because of its single-center, retrospective cohort design, the data collection may have created a degree of selection and information bias, and our results need to be validated in future studies. Second, genetic testing was not routinely available for the diagnosis and treatment procedures, and we did not detect differences in genotypes between isolated DCM and DCM with LV hypertrabeculation. Finally, the diagnosis of DCM and LV hypertrabeculation can be challenging. Etiological diagnosis of cases may not be included in the analysis owing to the lack of genetic testing, and there are multiple diagnostic criteria for LV hypertrabeculation. Thus, we used the Jenni criteria via echocardiography and checked by two independent cardiologists. 

## 5. Conclusions

We found that DCM with LV hypertrabeculation had a higher early (first 5 years) risk of MACEs. Especially for male patients, the presence of LV hypertrabeculation phenotypes might be an important clue for identifying high-risk DCM patients. These findings could help provide individualized management and facilitate precise treatment of DCM patients.

## Figures and Tables

**Table 1:** Clinical characteristics of dilated cardiomyopathy patients.

Characteristics	Total (*n* = 450)	Isolated DCM (*n* = 353)	DCM with LV Hypertrabeculation (*n* = 97)	*p* Value
Demographic and clinical features				
Age, years	48.1 ± 16.0	48.4 ± 15.9	47.0 ± 16.4	0.44
Distribution of age, No. (%)				
<60 years	339 (75.3)	265 (78.2)	74 (76.3)	0.81
≥60 years	111 (24.7)	88 (24.9)	23 (23.7)	
Male, No. (%)	305 (67.8)	245 (80.3)	60 (61.9)	0.16
Body mass index, kg/m^2^	24.8 ± 4.7	25.2 ± 4.8	23.4 ± 3.9	0.001
NYHA functional class, No. (%)				
I or II	252 (56.0)	198 (56.1)	54 (55.7)	0.94
III or IV	198 (44.0)	155 (43.9)	43 (44.3)	
Medical history, No. (%)				
Hypertension	152 (33.8)	131 (37.1)	21 (21.6)	0.01
Diabetic mellitus	91 (20.2)	81 (22.9)	10 (10.3)	0.02
Hyperlipidemia	167 (37.1)	129 (36.5)	38 (39.2)	0.69
Coronary heart disease	40 (8.9)	32 (9.1)	8 (8.2)	0.73
Current smoking, No. (%)	207 (46.1)	167 (47.4)	40 (41.2)	0.25
Current drinking, No. (%)	209 (46.4)	169 (47.9)	40 (41.2)	0.25
Electrocardiography				
Atrial fibrillation	83 (18.4)	71 (20.1)	12 (12.4)	0.08
Left bundle branch block	82 (18.2)	58 (16.4)	24 (24.7)	0.06
Ventricular tachycardia	6 (1.3)	5 (1.4)	1 (1.0)	0.77
ST-T changes	195 (43.3)	166 (47.0)	29 (29.9)	0.01
Echocardiography features				
LVEDD, mm	66.4 ± 8.5	66.0 ± 8.4	67.8 ± 8.7	0.06
LVESD, mm	56.8 ± 8.5	56.5 ± 8.4	58.0 ± 8.8	0.13
LVEF, %	29.6 ± 7.5	29.6 ± 7.5	29.6 ± 7.8	0.97
IVS, mm	8.0 ± 1.6	8.0 ± 1.6	7.7 ± 1.6	0.13
Moderate to severe MR, No. (%)	150 (33.3)	114 (32.3)	36 (37.1)	0.37
Laboratory examinations				
Lipids profiles, mmol/L				
Total cholesterol	4.17 (3.42–4.83)	4.25 (3.50–4.89)	4.32 (3.37–4.77)	0.94
Triglycerides	1.22 (0.85–1.86)	1.23 (0.88–1.93)	1.17 (0.80–1.74)	0.49
HDL-C	0.97 ± 0.29	0.97 ± 0.29	1.00 ± 0.29	0.32
LDL-C	2.71 ± 0.88	2.68 ± 0.89	2.70 ± 0.89	0.85
Creatinine, μmol/L	87.9 ± 37.0	86.5 ± 31.0	92.8 ± 33.3	0.14
Ln(NT-proBNP), ng/L	7.71 ± 1.32	7.71 ± 1.29	7.69 ± 1.46	0.92
Missing data	57 (12.7)	43 (12.2)	14 (14.4)	0.56
Medications, No. (%)				
ACEI/ARB/ARNI	396 (88.0)	316 (89.5)	80 (82.5)	0.06
β-blocker	418 (93.1)	330 (93.8)	88 (90.7)	0.30
Diuretics	330 (73.3)	263 (74.5)	67 (69.1)	0.28
Statins	142 (31.6)	116 (32.9)	26 (26.8)	0.26
Antiplatelet drugs	132 (29.3)	111 (31.4)	21 (21.6)	0.06
Anticoagulant drugs	110 (24.4)	84 (23.8)	26 (26.8)	0.73

Abbreviations: DCM, dilated cardiomyopathy; LV, left ventricular; NYHA, New York Heart Association; LVEDD, left ventricular end-diastolic diameter; LVESD, left ventricular end-systolic diameter; LVEF, left ventricular ejection fraction; IVS, interventricular septum; MR, mitral regurgitation; HDL-C, high-density lipoprotein cholesterol; LDL-C, low-density lipoprotein cholesterol; NT-proBNP, N-terminal pro-brain natriuretic peptide; ACEI, angiotensin-converting enzyme inhibitor; ARB, angiotensin receptor blocker; ARNI, angiotensin receptor neprilysin inhibitor.

**Table 2:** Univariable and multivariable Cox regression analyses of risk factors among dilated cardiomyopathy patients for major adverse cardiac events.

Variables	Univariable Analysis		Multivariable Analysis	
	HR (95%CI)	*p* Value	HR (95%CI)	*p* Value
Age, per 10-year increment	1.09 (1.00–1.18)	0.04	1.04 (0.95–1.15)	0.39
Male	1.11 (0.85–1.46)	0.45	-	-
BMI, per 1 Kg/m^2^ increment	0.98 (0.96–1.02)	0.32	-	-
NYHA class ≥ III	1.40 (1.09–1.81)	0.01	1.02 (0.73–1.43)	0.90
Current smoking	0.94 (0.73–1.21)	0.64	-	-
Current drinking	0.89 (0.69–1.14)	0.35	-	-
Electrocardiography				
Atrial fibrillation	1.08 (0.79–1.48)	0.35	-	-
Left bundle branch block	0.87 (0.63–1.21)	0.41	-	-
Ventricular tachycardia	1.33 (1.02–1.71)	0.03	1.31 (0.97–1.78)	0.08
ST-T changes	1.17 (0.91–1.51)	0.22	-	-
Echocardiography features				
LVEDD, per 5 mm increment	1.09 (1.02–1.17)	0.01	1.02 (0.78–1.32)	0.91
LVESD, per 5 mm increment	1.11 (1.03–1.19)	0.004	1.06 (0.97–1.17)	0.18
LVEF, per 1 SD increase	0.92 (0.82–1.04)	0.20	-	-
IVS, per 1 SD increase	0.99 (0.86–1.113)	0.85	-	-
Moderate to severe MR	1.77 (1.37–2.29)	<0.001	1.58 (1.16–2.16)	0.004
Laboratory examinations				
Total cholesterol, per 1 mmol/L	1.03 (1.00–1.07)	0.09	-	-
Triglycerides, per 1 mmol/L	0.82 (0.70–0.95)	0.01	0.84 (0.70–1.02)	0.08
HDL-C, per 1 mmol/L	0.84 (0.52–1.35)	0.47	-	-
LDL-C, per 1 mmol/L	0.91 (0.78–1.06)	0.23	-	-
Creatinine, per 1 μmol/L increment	1.00 (1.00–1.01)	0.14	-	-
Ln(NT-proBNP), per 1 ng/L increment	1.33 (1.19–1.48)	<0.001	1.26 (1.11–1.42)	<0.001
Medications				
ACEI/ARB/ARNI	0.69 (0.49–0.98)	0.04	0.78 (0.50–1.22)	0.28
β-blocker	0.50 (0.33–0.77)	0.001	0.60 (0.35–1.02)	0.06
Diuretics	1.63 (1.19–2.22)	0.002	1.15 (0.77–1.71)	0.50
Statins	1.00 (0.76–1.31)	0.99	-	-
Antiplatelet drugs	1.19 (0.91–1.54)	0.20	-	-
Anticoagulant drugs	1.04 (0.77–1.39)	0.81	-	-

Abbreviations: BMI, body mass index; NYHA, New York Heart Association; LVEDD, left ventricular end-diastolic diameter; LVESD, left ventricular end-systolic diameter; LVEF, left ventricular ejection fraction; IVS, interventricular septum; MR, mitral regurgitation; HDL-C, high-density lipoprotein cholesterol; LDL-C, low-density lipoprotein cholesterol; NT-proBNP, N-terminal pro-brain natriuretic peptide; ACEI, angiotensin-converting enzyme inhibitor; ARB, angiotensin receptor blocker; ARNI, angiotensin receptor neprilysin inhibitor.

**Table 3:** The incidence of cardiovascular events in different subtypes of dilated cardiomyopathy.

	Isolated DCM (*n* = 353)	DCM with LV Hypertrabeculation (*n* = 97)	Model 1		Model 2	
			HR (95%CI)	*p* Value	HR (95%CI)	*p* Value
Major adverse cardiac events						
No./Person-years	184/1611.7	60/451.7	1.17 (0.87–1.57)	0.29	1.25 (0.90–1.75)	0.19
Heart failure						
No./Person-years	156/1685.6	48/465.6	1.11 (0.80–1.54)	0.55	1.13 (0.777–1.67)	0.53
Major ventricular arrhythmias						
No./Person-years	21/1895.2	9/541.2	1.01 (0.41–2.51)	0.99	0.93 (0.32–2.65)	0.89
Left ventricular assist device implantation						
No./Person-years	9/1970.8	2/554.7	0.79 (0.47–1.33)	0.37	0.41 (0.05–3.75)	0.43
Heart transplantation						
No./Person-years	8/1927.9	1/551.3	0.36 (0.04–2.90)	0.34	0.25 (0.03–2.10)	0.20
All-cause death						
No./Person-years	82/1982.5	17/547.0	0.79 (0.47–1.33)	0.37	0.98 (0.56–1.70)	0.93
Cardiovascular death						
No./Person-years	82/1982.5	15/537.8	0.76 (0.44–1.33)	0.34	0.97 (0.54–1.75)	0.93

Model 1 adjusted age and sex; model 2 further adjusted BMI, NYHA functional class, medical history of hypertension and diabetes, ST-T changes, moderate to severe MR, and Ln(NT-proBNP).

## Data Availability

The authors are willing to provide a de-identified copy of the data upon reasonable request.

## Supplementary Materials

The following supporting information can be downloaded at https://www.mdpi.com/article/10.3390/jcdd11070220/s1, Table S1: Clinical characteristics of included and excluded patients; Table S2: The incidence of major adverse cardiac events and heart failure in different subtypes of dilated cardiomyopathy.
