# Supplementary material for: Characterization and Long-Term Prognosis of Patients with Different Phenotypes of Dilated Cardiomyopathy
Source: J Cardiovasc Dev Dis. 2024 Jul 12;11(7):220. doi: 10.3390/jcdd11070220 (PMC11277054; doi:10.3390/jcdd11070220)
Supplement: Supplementary file 1 [file jcdd-11-00220-s001.zip › jcdd-3019127-supplementary.pdf]

Supplemental material

Table S1. Clinical characteristics of included and excluded patients

Table S2. The incidence of major adverse cardiac events and heart failure in different subtypes of dilated cardiomyopathy

Table S1. Clinical characteristics of included and excluded patients

Characteristics	Included (n=450)	Excluded (n = 34)	*P* value
**Demographic and clinical features**			
Age, yrs	48.1 ± 16.0	47.0 ± 16.4	0.44
Distribution of age, No. (%)			
<60 years	339 (75.3)	29 (85.3)	0.22
≥ 60 years	111 (24.7)	5 (14.7)	
Male, No. (%)	305 (67.8)	20 (62.5)	0.56
Body mass index, kg/m^2	24.8 ± 4.7	23.4 ± 3.9	0.001
NYHA functional class, No. (%)			
I or II	252 (56.0)	16 (50.0)	0.58
III or IV	198 (44.0)	16 (50.0)	
Medical history, No. (%)			
Hypertension	152 (33.8)	9 (28.1)	0.57
Diabetic mellitus	91 (20.2)	6 (18.8)	0.84
Hyperlipidemia	167 (37.1)	13 (40.6)	0.87
Coronary heart disease	40 (8.9)	2 (6.3)	0.81
Current smoking, No. (%)	207 (46.1)	10 (32.3)	0.51
Current drinking, No. (%)	209 (46.4)	8 (25.9)	0.06
**Electrocardiography**			
Atrial fibrillation	83 (18.4)	3 (10.3)	0.27
Left bundle branch block	82 (18.2)	1 (3.4)	0.04
Ventricular tachycardia	6 (1.3)	0	0.84
ST-T changes	195 (43.3)	12 (41.4)	0.84
**Echocardiography features**			
LVEDD, mm	66.4 ± 8.5	63.9 ± 9.3	0.12
LVESD, mm	56.8 ± 8.5	53.8 ± 9.3	0.06
LVEF, %	29.6 ± 7.5	34.0 ± 6.9	0.002
IVS, mm	8.0 ± 1.6	8.6 ± 1.8	0.03
Moderate to severe MR, No. (%)	150 (33.3)	10 (32.3)	0.90
**Laboratory examinations**			
Lipids profiles, mmol/L			
Total cholesterol	4.17 (3.42–4.83)	4.25 (3.58–5.04)	0.83
Triglycerides	1.22 (0.85–1.86)	1.23 (0.76–1.85)	0.91
HDL-C	0.97 ± 0.29	1.03 ± 0.30	0.32
LDL-C	2.71 ± 0.88	2.84 ± 1.08	0.33
Creatinine, μmol/L	87.9 ± 37.0	80.0 ± 23.6	0.25
Ln(NT-proBNP), ng/L	7.71 ± 1.32	7.11 ± 1.51	0.02

Abbreviations: DCM, dilated cardiomyopathy; LVNC, left ventricular noncompaction; NYHA, New York Heart Association; LVEDD, left ventricular end-diastolic diameter; LVESD, left ventricular end-systolic diameter; LVEF, left ventricular ejection fraction; IVS, interventricular septum; MR, mitral regurgitation; HDL-C, high-density lipoprotein cholesterol; LDL-C, low-density lipoprotein cholesterol; NT-proBNP, N-terminal pro-brain natriuretic peptide.

Table S2. The incidence of major adverse cardiac events and heart failure in different subtypes of dilated cardiomyopathy

	Isolated DCM	DCM with LVNC	Model 1		Model 2	
			HR (95%CI)	*P* value	HR (95%CI)	*P* value
**Follow-up year 0-5**						
Major adverse cardiac events						
No. /Person-years	121/500.8	38/93.4	1.70 (1.17-2.47)	0.005	1.83 (1.21-2.77)	0.004
Heart failure						
No. /Person-years	98/567.5	31/104.6	1.72 (1.14-2.60)	0.01	1.50 (0.96-2.34)	0.07
**Follow-up year >5**						
Major adverse cardiac events						
No. /Person-years	63/1110.9	22/358.3	1.25 (0.75-2.08)	0.39	1.51 (0.80-2.84)	0.20
Heart failure						
No. /Person-years	58/1118.1	17/361.0	0.92 (0.52-1.61)	0.76	0.94 (0.46-1.93)	0.43

Model 1 adjusted age and sex;

Model 2 further adjusted BMI, NYHA class, medical history of hypertension and diabetes, ST-T changes, moderate to severe MR, and Ln(NT-proBNP).
